# ﻿*Actinostephanus* (Gesneriaceae), a new genus and species from Guangdong, South China

**DOI:** 10.3897/phytokeys.193.80715

**Published:** 2022-03-22

**Authors:** Fang Wen, Zi-Bing Xin, Xin Hong, Lei Cai, Xiao-Yun Chen, Jun-Jie Liang, Hui-Feng Wang, Stephen Maciejewski, Yi-Gang Wei, Long-Fei Fu

**Affiliations:** 1 Guangxi Key Laboratory of Plant Conservation and Restoration Ecology in Karst Terrain, Guangxi Institute of Botany, Guangxi Zhuang Autonomous Region and Chinese Academy of Sciences, CN-541006 Guilin, China; 2 Gesneriad Committee of China Wild Plant Conservation Association, National Gesneriaceae Germplasm Bank of GXIB, Gesneriad Conservation Center of China (GCCC), Guilin Botanical Garden, Guangxi Zhuang Autonomous Region and Chinese Academy of Sciences, CN-541006 Guilin, China; 3 School of Resources and Environmental Engineering, Anhui University, Hefei, CN–230601, Anhui Province, China; 4 Yunnan Key Laboratory for Integrative Conservation of Plant Species with Extremely Small Populations, Kunming Institute of Botany, Chinese Academy of Sciences, CN-650201, Kunming, Yunnan Province, China; 5 Key Laboratory for Plant Diversity and Biogeography of East Asia, Kunming Institute of Botany, Chinese Academy of Sciences, CN-650201, Kunming, Yunnan Province, China; 6 Management Office, Guangdong Enping Qixingkeng Provincial Nature Reserve, CN-529400, Enping, China; 7 Guangzhou Linfang Ecology Co., Ltd., Guangzhou, CN-510520, Guangdong Province, China; 8 The Gesneriad Society, 2030 Fitzwater Street, Philadelphia, PA. 19146-1333 USA

**Keywords:** *Boeica*, Didymocarpoideae, flora of China, IUCN, *Leptoboea*, phylogeny

## Abstract

*Actinostephanus*, a new genus from southern China, is described and colorfully illustrated with a single species, *A.enpingensis*. This new genus is morphologically most similar to *Boeica* and *Leptoboea*, nevertheless, it can be easily distinguished from the latter two by the following characteristics, such as leaves in whorls of three, all closely clustered at the top; corolla bowl-shaped, 5-lobed, actinomorphic; capsule hard, oblong-ovoid, short, 3–4 mm long, densely appressed villous, wrapped by persistent densely pubescent calyx lobes, style persistent. The new genus and related genera were sequenced using the next-generation sequencing technique. The whole plastid genome of the new genus is 154, 315 – 154, 344 bp in length. We reconstructed phylogenetic trees using the dataset of 80 encoded protein genes of the whole plastid genome from 47 accessions based on ML and BI analyses. The result revealed that the new genus was recovering in a polytomy including *Boeica*, *Rhynchotechum*, and *Leptoboea* with strong support, congruent to the morphological evidence. A global conservation assessment was also performed and classifies *A.enpingensis* as Least Concern (LC). In addition, after a review of recently described species of Gesneriaceae, we propose that plant enthusiasts, especially Gesneriad fans, have been playing an increasingly important role in the process of new taxa-discoveries.

## ﻿Introduction

The family Gesneriaceae has been extensively studied since the 1970s in China. There have been 19 new genera from China discovered and published by Chinese taxonomists and botanical researchers before 2005, i.e., *Allostigma* W.T.Wang, *Boeicopsis* H.W.Li, *Briggsiopsis* K.Y.Pan, *Buxiphyllum* W.T.Wang & C.Z.Gao, *Calcareoboea* C.Y.Wu ex H.W.Li, *Chiritopsis* W.T.Wang, *Didymostigma* W.T. Wang, *Deltocheilos* W.T.Wang, *Dolicholoma* D.Fang & W.T.Wang, *Gyrocheilos* W.T.Wang, *Gyrogyne* W.T.Wang, *Hemiboeopsis* W.T.Wang, *Metabriggsia* W.T.Wang, *Paralagarosolen* Y.G.Wei, *Pseudochirita* W.T.Wang, *Schistolobos* W.T.Wang, *Thamnocharis* W.T.Wang, *Tumidinodus* H.W.Li, *Wentsaiboea* D.Fang & D.H.Qin (Wang 1981a, b; Li 1982, 1983; Wang 1983a, b, c, 1984a, b; Pan 1985; Fang and Qin 2004; Wei 2004). However, the confirmation of all the genera mentioned-above relied only on the significances of morphological characters. Some were subsequently canceled in the process of compiling Flora Reipublicae Popularis Sinicae (Vol. 69) and Flora of China (Vol. 18) (Wang et al. 1990, Wang et al. 1998). *Boeicopsis*, *Buxiphyllum*, *Schistolobos* and *Tumidinodus* were treated as synonyms of *Boeica* Clarke, *Paraboea* (Clarke), Ridley, *Opithandra* Burtt, and *Anna* Pellegr, respectively (Wang et al. 1990, Wang et al. 1998; Li and Wang 2005). In addition, *Opithandra* was merged into *Oreocharis* Benth. (Möller et al. 2011).

Apart from morphological data, recognizing and describing new taxa with molecular evidence will result in a more robust and rational taxa (Fu et al. 2021). The first Chinese paper to mention the molecular method, estimated a molecular phylogeny of the previous subfamily Cyrtandroideae using two DNA regions (Wang and Li 1998). However, the Chinese described new taxa of Gesneriaceae in China using molecular evidence starting around 2010. The first one was a new genus, *Litostigma* Y.G.Wei, F.Wen & M.Möller. It was confirmed and published using classical taxonomy, palynology, and phylogenetic analysis (Wei et al. 2010). Since then, some redefined genera, such as *Hemiboea* Clarke (Weber et al. 2011a), *Loxostigma* Clarke (Möller et al. 2014), *Oreocharis* (Möller et al. 2011), *Paraboea* (Clarke) Ridley (Puglisi et al. 2011), *Petrocodon* Hance (Weber et al. 2011b), *Primulina* Hance (Weber et al. 2011c), *etc.*, and newly divided or restored genera, for example, *Glabrella* Mich.Möller & W.H.Chen (Möller et al. 2014) and *Bournea* Oliv. (Chen et al. 2020), were confirmed by the molecular and morphological evidence.

In 2017, a plant enthusiast, Mr. Yi Huang, sent the authors some photos of a rare and distinct Gesneriaceae plant, and we considered it a new taxon but could not undertake further studies since no material was collected at that time. Coincidentally, in 2019, one of the authors, Mr. Hui-Feng Wang, collected this species while undertaking a field trip in southern Guangdong, China.

After a careful morphological comparison, we could not place it into any genus of Gesneriaceae despite it sharing some similarities with *Boeica* or *Leptoboea*. To better understand the generic placement of this species, molecular phylogenetic analysis was also performed. After consulting the relevant literature (Wang et al. 1998; Li and Wang 2005; Wei et al. 2010; Wei 2018; Wen et al. 2019) and the molecular evidence, we concluded that this new species was assignable to a new genus, *Actinostephanus* gen. nov.

## ﻿Materials and methods

### ﻿Ethics statement

The only known location where this new species was found and collected was in the Qixingkeng provincial natural reserve, Enping, Guangdong. Two authors, Ms. Xiao-Yun Chen and Mr. Jun-Jie Liang, are staff at this natural reserve. They helped us get specific permission to enter the reserve and collect specimens. Our field studies did not involve any endangered or protected species. Further, special permits to conduct this research were not required.

### ﻿Material collection

This new species/genus has been monitored in the field by staff from Qixingkeng provincial natural reserve and grown by the authors at the nursery of the Gesneriad Conservation Center of China (GCCC) and National Gesneriaceae Germplasm Resources Bank of Guangxi Institute of Botany (GXIB) since the plants were collected. We also collected leaf materials of this proposed new species, using silica gel to dry them in the field for DNA extraction.

### ﻿Genomic DNA extraction and sequencing

Leaf material for DNA extraction was dried using silica gel (Chase and Hills 1991). Genomic DNA was extracted using the CTAB protocol (Doyle and Doyle 1987). The total gDNA sample was sent to Majorbio (http://www.majorbio.com/, China) for library construction and next-generation sequencing. Short-insert (350 bp) paired-end read libraries preparation and 2 × 150 bp sequencing were performed on an Illumina (HiSeq4000) genome analyzer platform. Approximately 6 Gb of raw data for the new species was filtered using the FASTX-Toolkit to obtain high-quality clean data by removing adaptors and low-quality reads (http://hannonlab.cshl.edu/fastx_toolkit/download.html).

### ﻿Whole plastid genome assembly and annotation

Clean reads were paired and imported in Geneious Prime (Kearse et al. 2012). For plastid genome assembly, the clean reads were mapped to published plastid genome sequence (*Petrocodonjingxiensis*, Genbank accession number: NC_044477.1) as reference (Xin et al. 2019) using the Fine Tuning option in Geneious Prime (iterating set as 10 times) to exclude nuclear and mitochondrial reads. Then, de novo assembly was performed using Geneious Prime with a medium-low sensitivity setting to assemble the plastid genome sequence. The clean reads mapped the generated contigs using the Fine Tuning option in Geneious Prime (iterating set as 10 times) to fill gaps. Contigs could be concatenated using the Repeat Finder option implemented in Geneious Prime until a ~130 kb contig (including SSC, IR, and LSC) was built. The Inverted Repeat (IR) region was determined by the Repeat Finder option in Geneious Prime and was reverse copied to obtain the complete plastid genome. The annotation approach of the plastid genome was performed using CPGAVAS2 and PGA (Qu et al. 2019; Shi et al. 2019).

### ﻿Phylogenetic analyses

To confirm the placement of this new plant, we reconstructed phylogenetic trees using the dataset of 80 encoded protein genes of the whole plastid genome. The new plant is morphologically similar to *Boeica* or *Leptoboea*, both of which belong to Subtr. Leptobaeinae C.B.Clarke (Clarke 1883). Therefore, we sampled all genera within this subtribe except for *Championia* Gardner and representatives of other subtribes within the Gesneriaceae as in-group, and 11 species represented other families as out-group. Consequently, 11 accessions were newly generated, while 36 accessions were downloaded from NCBI. Sequences obtained from this study and their information are listed in Appendix I.

All gene sequences were extracted using the PhyloSuite v1.2.2 (Zhang et al. 2020) and aligned by MAFFT v7.4 (Katoh and Standley 2013). The aligned sequences were then concatenated with PhyloSuite v1.2.2 (Zhang et al. 2020). Phylogenetic analyses were conducted using maximum likelihood (ML) and Bayesian inferences (BI), respectively. For the BI tree, we employed MrBayes v3.2.6 (Ronquist et al. 2012) to obtain a maximum clade credibility (MCC) tree. The parameters set as follows: nst = 6, rates = invgamma. Bayesian inference was performed with the concatenate sequence, using two million generations, two runs, four chains, a temperature of 0.001, and 25% trees were discarded as burn-in, and trees were sampled every 1,000 generations. Then, we used ModelFinder (Kalyaanamoorthy et al. 2017) to find the best fit model for ML analysis and further conducted the ML tree using IQ-TREE v2.1.2 (Nguyen et al. 2014) with 1000 bootstrap replicates. Tree visualization was achieved in Figtree v1.4.3.

## ﻿Results

### ﻿Characteristics of the complete plastid genome and ribosomal DNA

**Figure 1.**
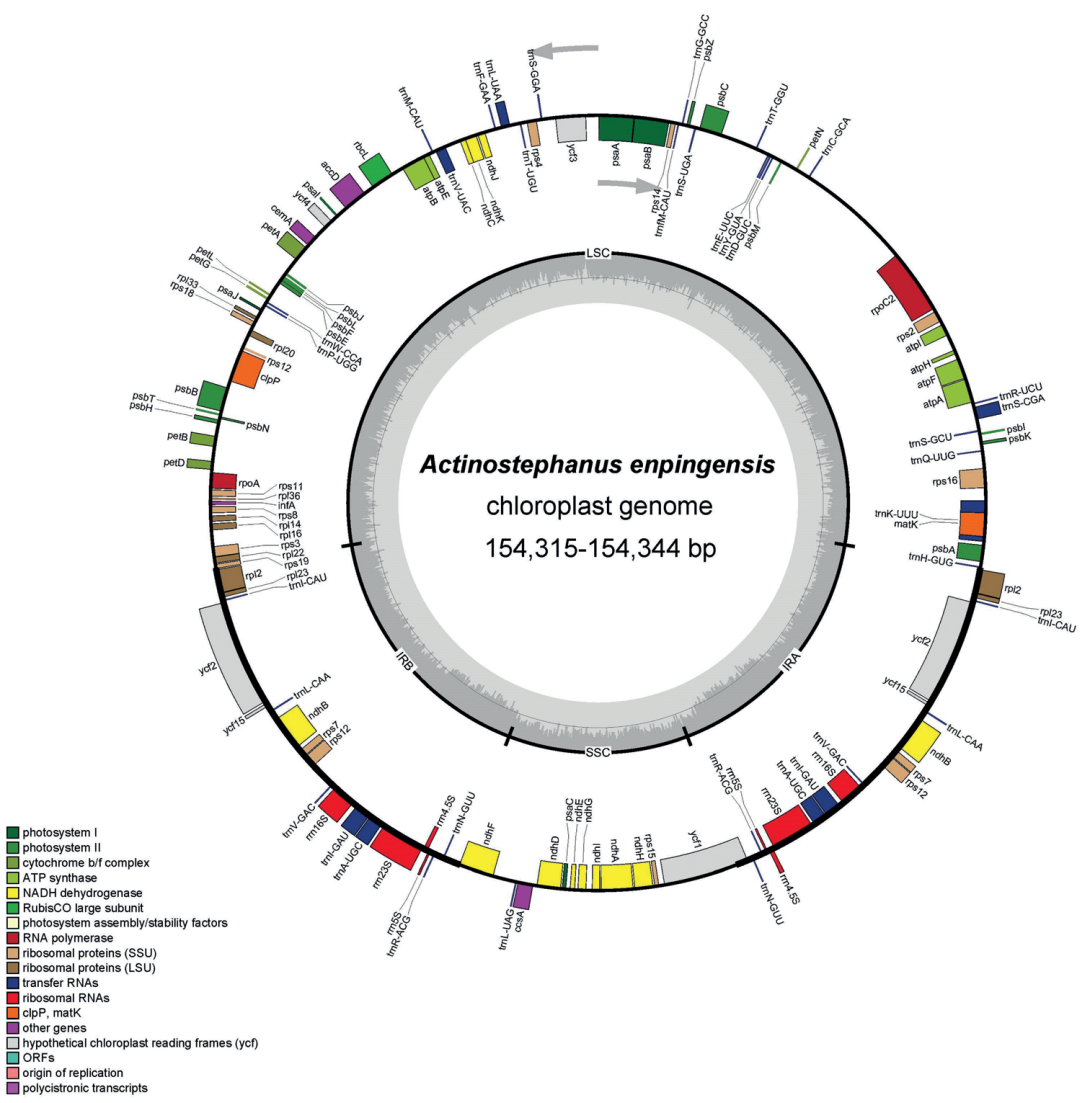
Plastid genome map of *Actinostephanusenpingensis*. The thick lines on the outer complete circle identify the inverted repeat regions (IRa and IRb). The innermost track of the plastome shows the GC content. Genes on the outside and inside of the map are transcribed in clockwise and counter directions, respectively.

The complete plastid genome of *Actinostephanusenpingensis* comprised 154,315 – 154,344 bp (Fig. 1). The characteristics and statistics of the plastid genome are summarized in Tables 1, 2.

**Table 1.** Summary of plastid genome of *Actinostephanusenpingensis*.

Characteristic	*Actinostephanusenpingensis*
Size (base pair, bp)	154,315-154,344
LSC length (bp)	85,450-85,479
SSC length (bp)	17,887-17,891
IR length (bp)	25,489-25,493
Number of genes	111-113
Protein-coding genes	77-79
rRNA genes	4
tRNA genes	30
LSC GC%	35.52%
SSC GC%	31.50%
IR GC%	43.20%

### ﻿Molecular phylogenetic studies

**Figure 2.**
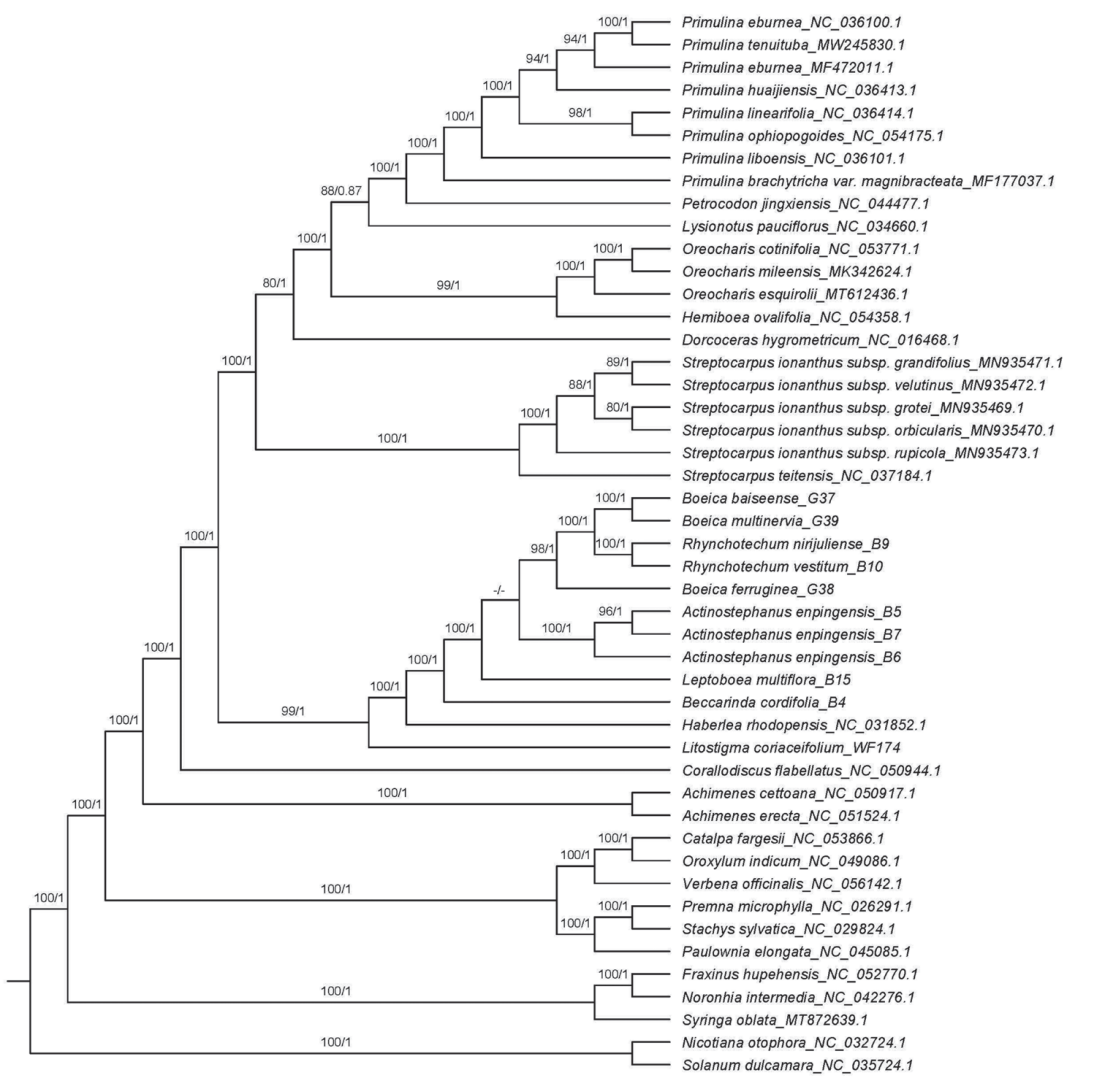
Phylogenetic tree of Gesneriaceae generated from maximum likelihood (ML) based on the dataset of whole-chloroplast protein-coding genes. Numbers on the branches indicate the bootstrap support (>70%) of the ML and the posterior probability (>0.8) of BI analyses.

BI and ML analyses of the dataset of 80 encoded protein genes of whole plastid genome resulted in the identical tree topologies that both indicate the three accessions of the new plant formed a strongly supported clade that was recovering in a polytomy including *Boeica*, *Rhynchotechum*, and *Leptoboea* in the clade of Subtr. Leptobaeinae (posterior probability (PP) = 1, bootstrap support (BS) = 100%) (Fig. 2).

**Table 2.** Genes encoded in plastid genome of *Actinostephanusenpingensis*.

Group of genes	Gene name
tRNA genes	*trnA*-*UGC* (×*2*), *trnC*-*GCA*, *trnD*-*GUC*, *trnE*-*UUC*, *trnF*-*GAA*, *trnG*-*GCC*, *trnH*-*GUG*, *trnI*-*CAU* (×*2*), *trnI*-*GAU* (×*2*), *trnK*-*UUU*, *trnL*-*CAA* (×*2*), *trnL*-*UAA*, *trnL*-*UAG*, *trnM*-*CAU*, *trnN*-*GUU* (×*2*), *trnP*-*UGG*, *trnQ*-*UUG*, *trnR*-*ACG* (×*2*), *trnR*-*UCU*, *trnS*-*CGA*, *trnS*-*GCU*, *trnS*-*GGA*, *trnS*-*UGA*, *trnT*-*GGU*, *trnT*-*UGU*, *trnV*-*GAC* (×*2*), *trnV*-*UAC*, *trnW*-*CCA*, *trnY*-*GUA*, *trnfM*-*CAU*
rRNA genes	*rrn16* (×*2*), *rrn23* (×*2*), *rrn4.5* (×*2*), *rrn5* (×*2*)
Ribosomal small subunit	rps16*, *rps2*, *rps14*, *rps4*, *rps18*, rps12** (×*2*), *rps11*, *rps8*, *rps3*, *rps19*, *rps7* (×*2*), *rps15*
Ribosomal large subunit	*rpl33*, *rpl20*, *rpl36*, *rpl14*, rpl16*, *rpl22*, rpl2* (×*2*), *rpl23* (×*2*)
DNA-dependent RNA polymerase	*rpoC2*, rpoC1*, *rpoB*, *rpoA*
Photosystem I	*psaB*, *psaA*, *psaI*, *psaJ*, *psaC*
Large subunit of rubisco	*rbcL*
Photosystem II	*psbA*, *psbK*, *psbI*, *psbM*, *psbC*, *psbZ*, *psbG*, *psbJ*, *psbL*, *psbF*, *psbE*, *psbB*, *psbT*, *psbN*, *psbH*
NADH dehydrogenase	*ndhJ*, *ndhK*, *ndhC*, ndhB* (×*2*), *ndhF*, *ndhD*, *ndhE*, *ndhG*, *ndhI*, ndhA*, *ndhH*
Cytochrome b/f complex	*petN*, *petA*, *petL*, *petG*, petB*, petD*
ATP synthase	*atpA*, atpF*, *atpH*, *atpI*, *atpE*, *atpB*
Maturase	*matK*
Translational initiation factor	*infA*
Subunit of acetyl-CoA carboxylase	*accD*
Envelope membrane protein	*cemA*
Protease	clpP**
C-type cytochrome synthesis	*ccsA*
Conserved open reading frames (ycf)	ycf3**, *ycf4*, *ycf2* (×*2*), *ycf1*, *ycf15* (×*2*)

Notes: Genes with one or two introns are indicated by one (*) or two asterisks (**), respectively. Genes in the IR regions are followed by the (×2) symbol.

### ﻿Ecology

Plants of the new taxon were primarily accessible in the Qixingkeng provincial natural reserve, growing on nearly vertical or steep slopes of montane yellow soil under tropical evergreen broad-leaved forest. Voucher specimens were made in the usual way (Bridson and Forman 1998) from some accessible plants that could be reached from the type locality. The conservation assessment was prepared following IUCN (2019).

### ﻿Taxonomic treatment

#### Subfam. Didymocarpoideae

##### ﻿ Actinostephanus

Taxon classificationPlantaeActiniariaActinodendridae

F.Wen, Y.G.Wei & L.F.Fu gen. nov.

F9E4B06C-604A-5CB5-9CA5-D9DB28B2EA7C

urn:lsid:ipni.org:names:77296131-1

###### Diagnosis.

*Actinostephanus* F.Wen, Y.G.Wei & L.F.Fu resembles two small genera, *Boeica* C.B.Clarke and *Leptoboea* Benth. according to the molecular evidence and some morphological data, but differs from the latter two by the following distinguishing characters: leaves in whorls of three, all closely clustered at the top; corolla bowl-shaped, 5-lobed, actinomorphic; capsule hard, oblong-ovoid, short, 3–4 mm long, densely appressed villous, wrapped by persistent densely pubescent calyx lobes, style persistent. The detailed distinguishing characters of this new genus and its congeners are listed in Table 1.

###### Type and only known species.

*Actinostephanusenpingensis* F.Wen, Y.G.Wei & Z.B.Xin, sp. nov.

###### Description.

Herbs, perennial, acaulescent, or forming elongated rhizome slightly fleshy growing after some years, rhizomes cylindrical, surface densely brown pubescent, fibrous root filiform, forming adventitious buds and plantlets in the middle or at the end of the fibrous root. Leaves all basal, whorls of three, sometimes opposite, all closely clustered at the top, forming a rosette, or clustered forming a rosette at the top of the rhizome after years of growth. Leaf-blades obovate elliptic, asymmetric, rarely symmetric, attenuate to base and base usually oblique, rarely aequilateral. Bracts 2. Calyx actinomorphic, 5-parted to the base. Corolla actinomorphic, bowl-shaped; tube very short, shallow bowl-shaped; limb quinquelobate, lobes equal. Stamens 4, separated, anthers dorsifixed, free, dehiscing longitudinally. Disc glabrous, margin crenulate. Ovary conical, stigma punctate. Capsule oblong-ovoid, appressed villous, wrapped by persistent calyx lobes, and the abaxial surfaces of calyx lobes covered densely pubescent. The number of seeds per capsule fewer. Seeds bigger, elliptic, both ends pointed.

###### Etymology.

The genus name, “*Actinostephanus*”, consists of two parts, both derived from the Greek. The front part, “*Actino*-” is derived from ἀκτῑ́ς (aktῑ́s, “ray, beam”), means radiating; the latter half, “-*stephanus*”, is derived from Στέφανος (Stéphanos, “crown”), is also closely associated in ᾰ̓́νθος (ánthos, “flower, blossom, bloom”), hints corolla. The combined Greek word-roots characterize the uncommon corolla characteristic of the new genus and species. The character of the corolla, in China’s Gesneriaceae, is rare. Only three species belonging to two genera were known to have actinoform corolla in China, namely *Bourneasinensis* Oliv., *B.leiophylla* (W.T.Wang) W.T.Wang & K.Y.Pan ex W.T.Wang and *Oreocharisesquirolii* H.Lév. before this new genus was discovered.

###### Vernacular name of the new genus.

Chinese mandarin: Fú Guàn Jù Tái Shǔ (辐冠苣苔属).

###### Distribution and habitat.

Endemic to Enping county, Guangdong province, China, under evergreen broadleaved forests in a montane mountain yellow soil area at 170–250 m altitude.

##### ﻿ Actinostephanus enpingensis

Taxon classificationPlantaeActiniariaActinodendridae

F.Wen, Y.G.Wei & Z.B.Xin sp. nov.

4ADCABF0-FDDB-5F31-BF2F-A8509E258465

urn:lsid:ipni.org:names:77296132-1

Figs 3 , 4

###### Type.

China. Guangdong province, Enping city, Naji town, Qixingkeng provincial natural reserve, growing on the cliffs or slopes of montane yellow soil and sandy loam near a stream. 22°11.1808'N, 112°5.841'E, ca. 153 m, *Chen Xiaoyun & Liang Junjie 210519-01* (holotype: IBK!, isotypes IBK!).

**Figure 3.**
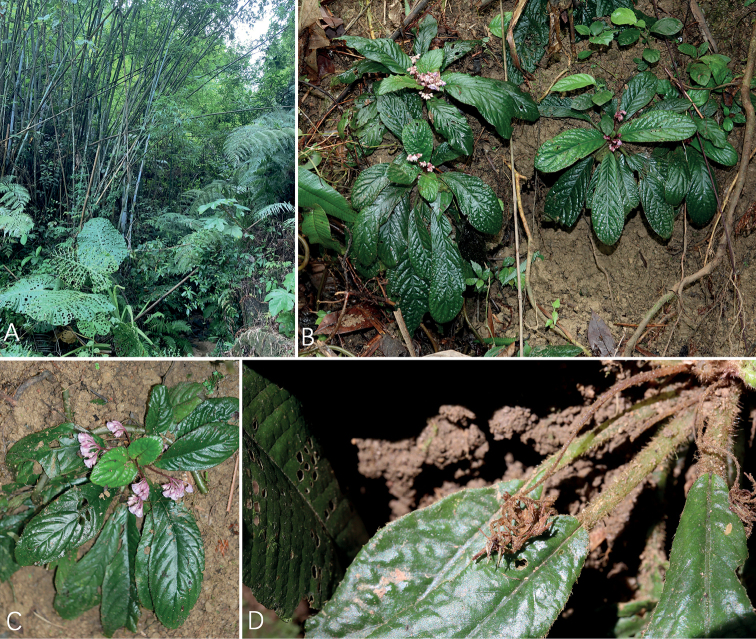
Photos of *Actinostephanus* F.Wen, Y.G.Wei & L.F.Fu gen. nov. (*A.enpingensis* F.Wen, Y.G.Wei & Z.B.Xin sp. nov.), the individuals in natural habitat. **A** habitat **B** habit **C** plant in flowering **D** plant in fruiting.

###### Description.

Herbs, perennial, acaulescent, or forming elongated rhizome slightly fleshy after some years, rhizomes cylindrical, surfaces densely brown pubescent, 5–15 mm long, 3–8 mm in diam., fibrous root filiform, 4–6 cm long, up to 10 cm, forming adventitious buds and plantlets in the middle or at the end of fibrous root. Leaves all basal, whorls of three, sometimes opposite, all closely clustered at top forming rosette, or clustered forming rosette at the top of rhizome after years of growth, (8)9–(16)18 or more, subsessile or shortly petiolate, short petiole cylindrical, 6–15 × 3.5–4.8 mm, densely brown villous. Leaf blades chartaceous to thickly herbaceous, thin chartaceous when dried, obovate elliptic, occasionally obovate lanceolate, greenery to green, dark green after a year of growth, 7.5–15.0 × 3.5–6.0 cm, asymmetric, rarely symmetric, attenuate to base and base usually oblique, rarely aequilateral, apex rounded, obtuse to subacute, margin numerous crenulate, adaxial surfaces of young leaf blades sparsely puberulent, subsequently gradually deciduous, adaxial surfaces of mature leaf blades nearly glabrous, but abaxial surfaces of young and mature leaf blades pubescent, covered by long and obvious strigose hairs along the main and lateral veins; venation alternate along main vein, lateral veins 7–9 on each side of the midrib, main and lateral veins on adaxial surface obviously sunken and on abaxial surface distinctly prominent. Inflorescence dichasium, 4–8, axially, 8–14-flowered, rarely 4–5-flowered and occasionally more than 14, 1–2-branched; peduncle sturdier, 2.2–4.5 cm long, 1.0–1.5 mm in diam., brownish-green to brownish-red, densely upward short strigose, the hairs brownish red, occasionally pink to pinkish white. Bracts 2, brownish-green, opposite, linear-lanceolate, ca. 6.0 × 1.0 mm, adaxial surface appressed pubescent, abaxial surface puberulent, apex acute, margin entire; pedicel 4.0–9.0 mm long, ca. 1.0 mm in diam., pale brownish-green to green, pubescent. Calyx actinomorphic, 5-parted to the base, segments pinkishwhite, pale pinkish-purple to pale brownish-red, equal, lanceolate, 3.5–4.0 × 1.2–1.4 mm, apex acute, margin entire, abaxial surface puberulent, adaxial surface glabrous, but persistent in the fruiting period. Corolla pale purple to pale bluish-purple, actinomorphic, bowl-shaped, 4.0–5.0 mm long/high, 65–75 mm in diam., outside puberulent, inside nearly glabrous and sparsely very few glandular-puberulent; tube very short, 1.5–2.0 mm long/high, shallow bowl-shaped; limb quinquelobate, lobes equal, half elliptic, the major axis ca. 3.5 mm long, the minor axis ca. 1.2 mm long, apex cambered, margin revolute. Stamens 4, separated, filaments nearly slender cylinder, glabrous, two longer and two shorter, longer pairs ca. 1.5 mm long, shorter pairs ca. 1.0 mm long, the four adnate to the base of corolla tube, anthers dorsifixed, free, cordate, yellowish-brown to pale greenish-brown, ca. 1.0 mm height, ca. 1.0 mm across at the bottom of the cordate shape, dehiscing longitudinally, glabrous. Disc wax yellow, ca. 1.0 mm high, glabrous, smooth, margin crenulate. Pistil 4.5–4.8 mm long, ovary pale pink, conical, sparsely inconspicuously puberulent, ca. 0.9 mm long, ca. 1.0 mm across at bottom, style translucent to white, 3.8–4.0 mm long, stigma punctate, yellow. Capsule oblong-ovoid, 4.5–5.0 mm long, 1.2–1.3 mm across, appressed villous, wrapped by persistent calyx lobes and the abaxial surfaces of calyx lobes densely pubescent; capsule hard when mature, style usually persistent, rarely dehiscent, occasionally split into 4-valves. The number of seeds per capsule fewer than 100, only 50–80, the macroaxis of seeds bigger, ca. 0.5 × 0.3 mm, brownish-black, not appendant, elliptic, both ends pointed.

**Figure 4.**
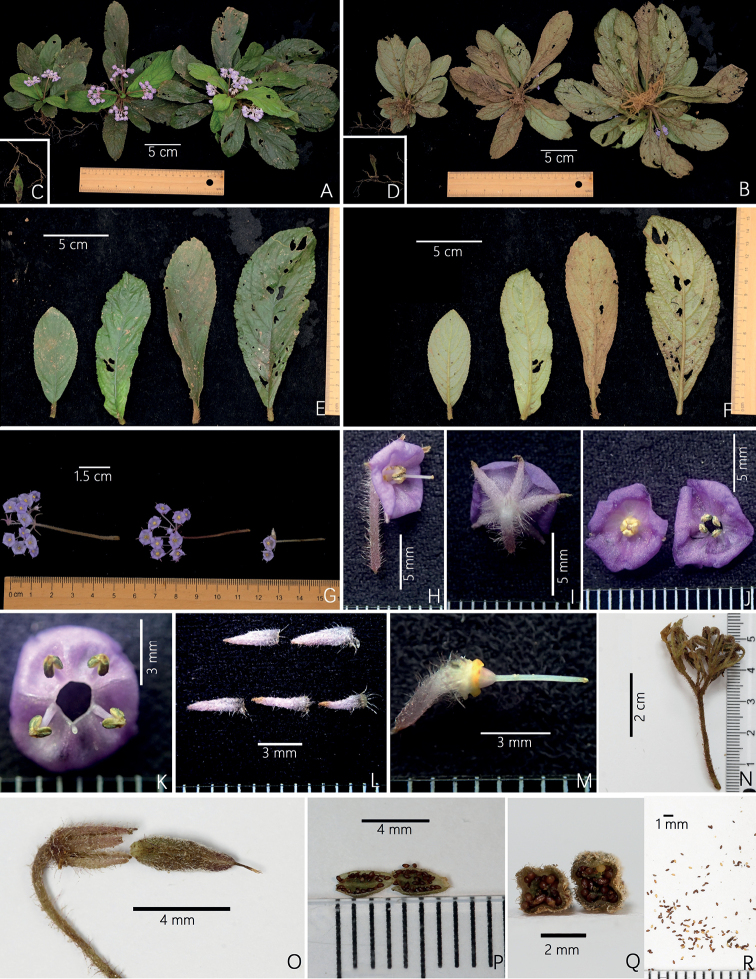
Photos of *Actinostephanus* F.Wen, Y.G.Wei & L.F.Fu gen. nov., the individuals in natural habitat. *A.enpingensis* F.Wen, Y.G.Wei & Z.B.Xin sp. nov. **A** top view of plant **B** upward view of plant for showing root system **C** top view of bud at the end of root **D** upward view of bud at the end of root **E** adaxial surfaces of leaves **F** abaxial surfaces of leaves **G** cymes **H** lateral view of flower **I** posterior view of flower **J** frontal view of corolla **K** stamens and staminodes **L** abaxial surfaces of calyx lobes **M** pistil **N** infructescence **O** capsule and persistent calyx lobes **P** opened capsule **Q** cross-section of capsule **R** seeds.

###### Preliminary conservation status.

Based on the result of our joint field surveys in the type locality and adjacent regions, the EOO and AOO of *Actinostephanusenpingensis* are about 79.5 km^2^ and 0.1 km^2^, respectively. So far, only one population of this species has been recorded along the local stream in the Qixingkeng provincial natural reserve, Enping city, Guangdong province, southern China, but we believe that more *A.enpingensis* populations can be found in the hills of Enping and its adjacent counties. If that is the case, the Extent of Occurrence (EOO) and Area of Occupancy (AOO) of this species will increase. Because the flowers and leaves of this species are inconspicuous, and after learning from some local people that it has no known medicinal value we feel that this species faces little risk. Moreover, almost all of these plants are growing in the protected areas of this reserve so that the species are well protected. According to the Guidelines for Using the IUCN Red List Categories and Criteria (IUCN 2019), we access this taxon as a Least Concern species (LC).

## ﻿Discussion

Our phylogenetic studies revealed that the new plant fell into Subtr. Leptoboeinae C.B.Clarke (PP = 1, BS = 100%). This subtribe belongs to Tribe Trichosporeae Nees, Subfamily Didymocarpoideae. At present, six genera have been included in this subtribe (Möller et al. 2017). Although their morphologies from different genera of this Subtribe are heterogeneous, several characters, such as the absence of large flowers, the inconspicuous to capitate stigma, straight but no-twisted fruits, and commonly 4-valved and dehiscent capsules or fleshly berries were concluded as common ones (Weber at al. 2013, Weber at al. 2020). The new plant is morphologically congruent with these characters that further indicate the monophyly of Subtr. Leptoboeinae. Within Subtr. Leptoboeinae, the new plant, was recovering in a polytomy including *Boeica*, *Rhynchotechum*, and *Leptoboea*. Two *Boeica* spp. are most closely related to *Rhynchotechum*, and both sisters to the type species of *Boeica* (*B.furruginea*) indicated that *Boeica* is not monophyletic. This relationship was congruent with previous studies (Yang et al. 2020). Expanding the sampling, and exploring key characters, is needed to re-define the *Boeica*. Despite this, the new genus is morphologically similar to these genera based on phyllotaxis and inflorescence cyme. However, it can be easily distinguished by corolla bowl-shaped, 5-lobed, actinomorphic, capsule densely appressed villous, wrapped by persistent densely pubescent calyx lobes and style persistent (Table 3). The most distinct characters of the new plant are its actinomorphic corolla, tiny fruit hard when mature, rarely dehiscent, occasionally split into 4-valves, style usually persistent, which are likely to be derived characters or autapomorphies. We, therefore, based on the molecular and morphological evidence, treat it as a new genus, namely, *Actinostephanus*.

**Table 3.** Comparison of morphological characters of *Actinostephanus*, *Boeica* and *Leptoboea*.

Characters	*Actinostephanus*	*Boeica*	*Leptoboea*
**Habit**	perennial herb but acaulescent, or elongated rhizome slightly fleshy growing after some years	subshrub, or perennial herb	Subshrub
**Stem**	Acaulescent	erect aerial stem, sometimes stolon, stem more or less lignified	erect aerial stem, lignified
**Stolon and root system**	no stolon, fibrous root filiform, at the end of root with ability for cloning	stolon or no stolon; roots no fecundity	no stolon; roots no fecundity
**Leaf**	whorls of three, all closely clustered at top	alternative	branches and leaves opposite, usually clustered at annual shoots
**Inflorescence**	cyme, corymbose, 1- or 2-branched; peduncle sturdier	cyme, multi-branched, occasionally no-branched; peduncle sturdier	cyme, corymbose, multi-branched; peduncle and pedicel slender similar to filiform
**Corolla**	actinomorphic, bowl-shaped, limb and tube nearly isometric, lobes deflexed	campanulate, corolla tube shorter than limb; limb slightly bilabiate, 5-lobed, lobes equal or slightly unequal	campanulate, small; limb slightly bilabiate, 5-lobed, lobes nearly equal
**Capsule**	oblong-ovoid, short, appressed villous, wrapped by persistent calyx lobes, and the calyx lobes also outside covered densely pubescent; hard when mature, style usually persistent, rarely dehiscent, occasionally split into 4-valves	linear, long, glabrous, apex pointed, style no persistent	long linear, long, glabrous, style no persistent
**Seed**	the number of seeds per capsule fewer, only 50–80	the number of seeds per capsule numerous, hundreds	the number of seeds per capsule numerous, hundreds

The high levels of plant species diversity and endemism in southern and southwestern China are more and more renowned, especially in karst regions. Nevertheless, it is evident that the geographic accessibility of those mountainous areas (including townships, villages, and surrounding regions) has been hindered by terrible transport problems. It also seriously affected the understanding of plant diversity in South and Southwest China. But with the fast development of the Chinese economic and construction systems, more and more road construction projects are being carried out, forming a relatively completed road transportation system in China. Thus, there are more opportunities to discover many taxa new to science. The people have easy access to those places that were difficult to reach in past decades.

Not only do more and more taxonomists focus on the biodiversity of Gesneriaceae in China, but plant enthusiasts are also making an enormous contribution to help botanists discover rare and new Gesneriads. As previously mentioned, Mr. Yi Huang, a plant enthusiast, found the interesting *Boeica*-like species of Gesneriaceae in South China, and he offered this critical information to us. Thus, we will be more conscious of this uncertain species over the next few years. Therefore, we propose that plant enthusiasts, especially Gesneriad fans, are playing an increasingly important role in the process of new taxa-discoveries.

Several new species, for example, *Primulinapapillosa* Z.B.Xin, W.C.Chou & F.Wen (Xin et al. 2021), *P.purpureokylin* F.Wen, Yi Huang & W.C.Chou, *P.niveolanosa* F.Wen, S.Li & W.C.Chou, *P.persica* F.Wen, Yi Huang & W.C.Chou (Li et al. 2019), were discovered by Mr. Wei-Chuen Chou, who is passionate about collecting *Primulina* species. He has also registered a number of new ornamental varieties by hybridization with the Gesneriad Society, the International Registration authority for Gesneriad horticultural variety. Other examples are *P.longii* (Z.Y.Li) Z.Y.Li and *P.leiyyi* F.Wen, Z.B.Xin & W.C.Chou; the scientific names paid homage to the discovers and collectors, Mr. Guang-Ri Long and Mr. Yu-Yang Lei (Li 2002; Li et al. 2019). Since 2011, at least 14 taxa in China (including those five new species above-mentioned) were discovered and published with the assistance of domestic Gesneriad enthusiasts (non-professionals/botanists/taxonomists, who are not associated with any university, institute or botanical garden), based on our statistics. They are *Primulinaspiradiclioides* Z.B.Xin & F.Wen (Xin et al. 2020a), P.hochiensisvar.ochroleuca F.Wen, Y.Z.Ge & Z.B.Xin (Ge et al. 2020), *P.anisocymosa* F.Wen, Xin Hong & Z.J.Qiu (Hong et al. 2019), *P.wuae* F.Wen & L.F.Fu (Li et al. 2017), *P.qintangensis* Z.B.Xin, W.C.Chou & F.Wen (Xin et al. 2020b), *P.titan* Z.B.Xin, W.C.Chou & F. Wen (Xin et al. 2020c), P.bipinnatifidavar.zhoui (F.Wen & Z.B.Xin)W.B.Xu & K.F.Chung, *P.huangii* F.Wen & Z.B.Xin (Xin et al. 2018), *P.duanensis* F.Wen & S.L.Huang (Huang et al. 2015), *P.moi* F.Wen & Y.G.Wei (Zhou et al. 2015), *Oreocharisaimodisca* Lei Cai, Z.L.Dao & F.Wen, *O.longipedicellata* Lei Cai & F.Wen (Cai et al. 2020), *O.panzhouensis* Lei Cai, Y.Guo & F.Wen (Cai et al. 2019), *Didymocarpusdissectus* F.Wen, Y.L.Qiu, Jie Huang & Y.G.Wei (Wen et al. 2013).

## Supplementary Material

XML Treatment for Actinostephanus

XML Treatment for Actinostephanus enpingensis

## ﻿Appendix I

The following is a list of used samples that are ordered alphabetically by taxon with their GenBank accession number of whole plastid genome sequences respectively. The samples with newly generated sequences are listed with the complete voucher information.

*Actinostephanusenpingensis*_B5 Qixingkeng Provincial Nature reserve, Enping City, Guangdong, WF210730-02, IBK!, OM176663; *Actinostephanusenpingensis*_B6 Qixingkeng Provincial Nature reserve, Enping City, Guangdong, FZY210502-01, IBK!, OM176664; *Actinostephanusenpingensis*_B7 Qixingkeng Provincial Nature reserve, Enping City, Guangdong, WF210730-01, IBK!, OM176665; *Boeicabaiseense*_G37 Liujun village, Youjiang District, Baise City, Guangxi, WYG180520-05, IBK!, OM176669; *Boeicaferruginea*_G38 Pu Luong Nature Reserve, Thanh Hoa Province, Vietnam, WF21030328-01, IBK!, OM176670; *Boeicamultinervia*_G39 Jingping County, WF190814-01, IBK!, OM176671; *Beccarindacordifolia*_B4 Dulongjiang, Gongshan County, Yunnan, XZB2104, IBK!, OM176662; *Leptoboeamultiflora*_B15 Kauai National Forest, Thailand, WF180508-05, IBK!, OM176668; *Litostigmacoriaceifolium*_WF174 Maling Gorge, Xingyi City, Guizhou, FLF170420-01, IBK!, OM176672; *Rhynchotechumnirijuliense*_B9 Beibeng to Mihan, Medog County, Xizang, WF200910-06, IBK!, OM176666; *Rhynchotechumvestitum*_B10 Beibeng to Mihan, Medog County, Xizang, WF200910-22, IBK!, OM176667; *Achimenescettoana*NC_050917; *Achimeneserecta*NC_051524.1; *Catalpafargesii*NC_053866.1; *Chiritaeburnea*NC_036100.1; *Corallodiscusflabellatus*NC_050944.1; *Dorcocerashygrometricum*NC_016468.1; *Fraxinushupehensis*NC_052770.1; Haberlea rhodopensis NC_031852.1; Hemiboea ovalifolia NC_054358.1; *Lysionotuspauciflorus*NC_034660.1; *Nicotianaotophora*NC_032724.1; *Noronhiaintermedia*NC_042276.1; *Oreochariscotinifolia*NC_053771.1; *Oreocharisesquirolii*MT612436.1; *Oreocharismileensis*MK342624.1; *Oroxylumindicum*NC_049086.1; *Paulowniaelongata*NC_045085.1; *Petrocodonjingxiensis*NC_044477.1; *Premnamicrophylla*NC_026291.1; Primulinabrachytrichavar.magnibracteataMF177037.1; *Primulinaeburnea*MF472011.1; *Primulinahuaijiensis*NC_036413.1; *Primulinaliboensis*NC_036101.1; *Primulinalinearifolia*NC_036414.1; *Primulinaophiopogoides*NC_054175.1; *Primulinatenuituba*MW245830.1; *Solanumdulcamara*NC_035724.1; *Stachyssylvatica*NC_029824.1; Streptocarpusionanthussubsp.grandifoliusMN935471.1; Streptocarpusionanthussubsp.groteiMN935469.1; Streptocarpusionanthussubsp.orbicularisMN935470.1; Streptocarpusionanthussubsp.rupicolaMN935473.1; Streptocarpusionanthussubsp.velutinusMN935472.1; *Streptocarpusteitensis*NC_037184.1; *Syringaoblata*MT872639.1; *Verbenaofficinalis*NC_056142.1.
